# Sphingosine-1-Phosphate Receptor-2 Antagonists: Therapeutic Potential and Potential Risks

**DOI:** 10.3389/fphar.2016.00167

**Published:** 2016-06-21

**Authors:** Kira V. Blankenbach, Stephanie Schwalm, Josef Pfeilschifter, Dagmar Meyer zu Heringdorf

**Affiliations:** Institut für Allgemeine Pharmakologie und Toxikologie, Klinikum der Johann Wolfgang Goethe-UniversitätFrankfurt am Main, Germany

**Keywords:** sphingosine-1-phosphate, G-protein-coupled receptors, vascular tone, endothelial barrier, insulin sensitivity

## Abstract

The sphingosine-1-phosphate (S1P) signaling system with its specific G-protein-coupled S1P receptors, the enzymes of S1P metabolism and the S1P transporters, offers a multitude of promising targets for drug development. Until today, drug development in this area has nearly exclusively focused on (functional) antagonists at the S1P_1_ receptor, which cause a unique phenotype of immunomodulation. Accordingly, the first-in class S1P_1_ receptor modulator, fingolimod, has been approved for the treatment of relapsing-remitting multiple sclerosis, and novel S1P_1_ receptor (functional) antagonists are being developed for autoimmune and inflammatory diseases such as psoriasis, inflammatory bowel disease, lupus erythematodes, or polymyositis. Besides the S1P_1_ receptor, also S1P_2_ and S1P_3_ are widely expressed and regulate many diverse functions throughout the body. The S1P_2_ receptor, in particular, often exerts cellular functions which are opposed to the functions of the S1P_1_ receptor. As a consequence, antagonists at the S1P_2_ receptor have the potential to be useful in a contrasting context and different areas of indication compared to S1P_1_ antagonists. The present review will focus on the therapeutic potential of S1P_2_ receptor antagonists and discuss their opportunities as well as their potential risks. Open questions and areas which require further investigations will be emphasized in particular.

## Introduction

Sphingosine-1-phosphate (S1P), initially considered as an intermediate breakdown product of complex sphingolipid catabolism, is meanwhile acknowledged as important bioactive lipid (Maceyka et al., 2012; Kunkel et al., 2013; Blaho and Hla, 2014; Proia and Hla, 2015). S1P regulates crucial cellular functions such as proliferation and survival, migration, and adhesion (Maceyka et al., 2012; Blaho and Hla, 2014). Systemic and local gradients of S1P provide direction for migration and trafficking of various cell types (Olivera et al., 2013a; Nishi et al., 2014). Metabolically, S1P resides in a dynamically balanced equilibrium with sphingosine and ceramide (Hannun and Obeid, 2008; Newton et al., 2015). Ceramide, derived from *de novo* sphingolipid synthesis, from breakdown of glycosphingolipids or via the sphingomyelin cycle, can be converted reversibly into sphingosine, which in turn can be phosphorylated to S1P by the sphingosine kinases, SphK1 and SphK2 (Hannun and Obeid, 2008). S1P is a substrate of specific S1P phosphatases and non-specific lipid phosphate phosphatases which direct the equilibrium into the direction of sphingosine/ceramide (Hannun and Obeid, 2008). S1P can also be cleaved by S1P lyase, which generates hexadecenal and phosphoethanolamine and thus catalyzes an irreversible step of sphingolipid breakdown (Aguilar and Saba, 2012).

With regard to its complexity and to the multitude of options for pharmacological interventions, the S1P signaling system might well be compared to the adrenergic system. First of all, there are five specific G-protein-coupled S1P receptors, which are widely expressed and can act both in a redundant and in an antagonistic manner by coupling to distinct G-proteins (Blaho and Hla, 2014; Kihara et al., 2014), reminiscent of α_1_, α_2_, and β adrenergic receptors (Bylund et al., 1994; Alexander et al., 2015). Furthermore, the enzymes which catalyze the formation and degradation of S1P, as well as the specific and non-specific S1P transporters, represent promising drug targets (Meyer zu Heringdorf et al., 2013; Orr Gandy and Obeid, 2013; Nishi et al., 2014), reminiscent of the enzymes of catecholamine synthesis and degradation as well as the catecholamine transporters (Alexander et al., 2013). However, drug development targeting the S1P signaling system has focused almost exclusively on the S1P_1_ receptor until now (Roberts et al., 2013; Bigaud et al., 2014; Gonzalez-Cabrera et al., 2014). This is not least due to the success of the non-specific S1P receptor agonist, fingolimod, which has been approved for the treatment of multiple sclerosis in 2010/11 by the FDA and the EMA, respectively (Kihara et al., 2015). In brief, phosphorylated fingolimod, acting as a superagonist or functional antagonist at S1P_1_, causes internalization and degradation of the receptor, thereby rendering lymphocytes insensitive to the tissue-blood S1P gradient with the consequence of lymphopenia (Bigaud et al., 2014). Subsequent to the approval of fingolimod, novel S1P_1_ functional or competitive antagonists with improved properties are presently being developed (Meyer zu Heringdorf et al., 2013; Roberts et al., 2013; Bigaud et al., 2014; Gonzalez-Cabrera et al., 2014). While the development of sphingosine kinase and S1P lyase inhibitors has turned out to be not that straightforward (see e.g., Schnute et al., 2012; Deniz et al., 2015, and discussion in Meyer zu Heringdorf et al., 2013), the focus on G-protein-coupled S1P receptors other than S1P_1_ might be promising. Aside from causing immunosuppression, the effects of S1P_1_ receptor antagonism are rather undesirable: elevation of blood pressure, bronchial constriction, and on the long term a disturbance of the vascular endothelial barrier (Bigaud et al., 2014). Since the S1P_2_ receptor often acts contrarily to S1P_1_, S1P_2_ antagonists might turn up as promising tools for improving local blood flow in combination with tightening of the endothelial barrier, as anti-inflammatory and anti-fibrotic treatment options, and as potentially beneficial to treat the metabolic syndrome. The current review will discuss this therapeutic potential of S1P_2_ receptor antagonists and potential risks.

## Overview of G-Protein-Coupled S1P Receptors and Specific Features of S1P_2_

There are five specific G-protein-coupled receptors with a high affinity for S1P. According to the International Union of Basic and Clinical Pharmacology (IUPHAR) receptor nomenclature, they are named S1P_1_–S1P_5_ (human gene names S1PR1–S1PR5; Kihara et al., 2014). S1P_1-3_ are expressed nearly ubiquitously, whereas S1P_4_ is preferentially expressed in the hematopoietic system, and S1P_5_ is found in the white matter of the brain as well as in some other tissues (Blaho and Hla, 2014; Kihara et al., 2014, 2015; Pyne et al., 2015). S1P_1_ couples exclusively to G_i_, while S1P_2_ and S1P_3_ couple to G_i_, G_q_, and G_12/13_. S1P_4_ and S1P_5_ couple to G_i_ and G_12/13_. These receptors regulate cell growth and survival, migration, and adhesion. Thereby they orchestrate for example the circulation of lymphocytes, the formation and maturation of blood vessels, vascular tone and permeability, as well as heart rate (Blaho and Hla, 2014; Kihara et al., 2014). Furthermore, they play a role in inflammation, fibrosis, and cancer (Maceyka et al., 2012; Schwalm et al., 2013; Newton et al., 2015; Pyne et al., 2015).

The S1P_2_ receptor, also referred to as EDG-5, H218, AGR16, or lp_B2_, was originally cloned from rat aortic vascular smooth muscle cells (Okazaki et al., 1993) and later identified as high-affinity S1P receptor (Gonda et al., 1999). With a *K*_d_ of 27 nM and an EC_50_ in GTPγS binding of 3.8–8.9 nM, the affinity of S1P to S1P_2_ appears marginally lower than its affinity to S1P_1_ (*K*_d_ of 8 nM and EC_50_ in GTPγS binding 0.4–79; for review, see Spiegel, 2000; Kihara et al., 2014). S1P_2_ is coupled via G_i_, G_q_, and G_12/13_ to phospholipase C, [Ca^2+^]_i_ increases, activation of mitogen-activated protein kinases, and activation of Rho and/or Rho kinase (reviewed in Skoura and Hla, 2009; Adada et al., 2013; Blaho and Hla, 2014). Similar to S1P_1_, S1P_2_ can be internalized upon stimulation with agonists (see e.g., Ter Braak et al., 2011; Imeri et al., 2014). Mechanistically, this has been analyzed in zebrafish. The zebrafish miles apart mutant, S1P_2_ R150H, which impedes the migration of cardiac precursor cells to the midline, was shown to be constitutively desensitized and internalized (Burczyk et al., 2015). As this involved β-arrestin-2 and G-protein-coupled receptor kinase (GRK)-2, S1P_2_ R150H receptor functionality could be restored by reduced GRK2/3 expression (Burczyk et al., 2015).

S1P_2_ can activate Akt (Means et al., 2007), but in most cells, it inhibits Akt (Schüppel et al., 2008; Du et al., 2010; Michaud et al., 2010; Green et al., 2011; Japtok et al., 2015, and many other reports), and it can act pro- as well as anti-proliferative. Similarly, S1P_2_ can both activate and inhibit Rac (Okamoto et al., 2000; Sugimoto et al., 2003; Takashima et al., 2008; Rapizzi et al., 2009). S1P_2_ inhibited the phosphatidylinositol-3-kinase/Akt pathway by mediating Rho-dependent activation of the phosphoinositide phosphatase and tensin homolog deleted on chromosome 10 (PTEN) in fibroblasts and endothelial cells (Sanchez et al., 2005, 2007), but in macrophages, S1P_2_-mediated inhibition of Akt and migration were independent of PTEN (Michaud et al., 2010). Via G_12/13_ or G_q_ and activation of Rho/Rho kinase, in conjunction with inhibition of Rac, S1P_2_ exerts its eminent anti-migratory effect, important, e.g., for retention of B cells and follicular T helper cells in germinal centers (Green et al., 2011; Moriyama et al., 2014). The S1P_2_ receptor can furthermore regulate cytoskeleton organization via phosphorylation of ezrin/radixin/moesin (ERM) proteins (Gandy et al., 2013). This pathway appears to be involved in control of the endothelial barrier function and cancer cell invasion (Adyshev et al., 2011; Adada et al., 2015). Another signaling pathway recently discovered to be regulated by G-protein-coupled receptors including S1P_2_ is the Hippo pathway (Yu et al., 2012). S1P_2_, again via G_12/13_ and Rho, activated the transcription coactivators, YAP and TAZ (Miller et al., 2012; Yu et al., 2012). This occurred independently of the core Hippo pathway kinases (Miller et al., 2012), or via inhibition of LATS1/2 (Yu et al., 2012). The Hippo pathway regulates tissue proliferation and organ size, and plays a role in tumorigenesis (Zhang et al., 2015; Ye and Eisinger-Mathason, 2016).

The S1P_2_ receptor itself might be regulated by signals other than S1P. Thus, a recent report suggests that the well-known effect of S1P_2_ on neurite retraction (MacLennan et al., 2000; Toman et al., 2004) might be involved in neuronal Nogo-A signaling (Kempf et al., 2014). It was shown that Nogo-A-Δ20 directly activated S1P_2_ by binding to its extracellular loops 2 and 3 independently of the presence of S1P, and via S1P_2_/G_13_/RhoA inhibited neurite outgrowth and repressed synaptic plasticity (Kempf et al., 2014). The authors suggested that the S1P_2_ receptor is a multi-ligand receptor, regulated by both lipid and protein ligands (Kempf et al., 2014). Yet other potential agonists at S1P_2_ might be conjugated bile acids such as taurocholate (Studer et al., 2012). Several reports show that effects of conjugated bile acids, such as activation of ERK and Akt in primary rodent hepatocytes, and cell growth, survival, induction of cyclooxygenase (COX)-2 and formation of prostaglandin (PG)E_2_ in cholangiocarcinoma cells, are dependent on the S1P_2_ receptor, as they are blocked by the antagonist JTE-013 and by knocking down S1P_2_ (Studer et al., 2012; Liu et al., 2014, 2015). While these observations do not preclude that preparations of conjugated bile acids contain traces of S1P, taurocholate specifically induced ERK phosphorylation in S1P_2_ but not S1P_1_ overexpressing HEK-293 cells (data obtained with the other S1P receptors were not shown; Studer et al., 2012), thereby rebutting the contamination hypothesis. A direct interaction is furthermore supported by molecular modeling of taurocholate into S1P_2_ (Studer et al., 2012).

## S1P_2_ Receptor Pharmacology

S1P_2_ receptor pharmacology is still in its infancy as only few S1P_2_ selective compounds have been identified so far. These are still experimental tool compounds. Until recently, JTE-013 (Osada et al., 2002) was the only available S1P_2_ antagonist. Many functional studies have made use of this compound and thus, besides the results obtained with genetic approaches, the effects of JTE-013 have shaped our present view of the physiological roles of S1P_2_. However, JTE-013 has a low potency and lacks selectivity. This is illustrated by the fact that JTE-013 inhibited responses to S1P in S1P_2_ knockout mice, and it had effects in cells that had no S1P_2_ mRNA transcripts (Salomone et al., 2008; Li et al., 2012). Later, it was found that JTE-013 inhibited not only S1P_2_ but also the S1P_4_ receptor (Long et al., 2010). Furthermore, JTE-013 lacks stability *in vivo* (Li et al., 2015). Recently, AB1 has been developed as derivative of JTE-013 with improved stability *in vivo*, higher potency and better efficacy, but the selectivity of the compound is not well defined so far (Li et al., 2015). Other recently synthesized S1P_2_ antagonists require further characterization (Kusumi et al., 2015). Interestingly, a series of S1P_2_ agonists has also been described, of which at least CYM-5520 acted at an allosteric site (Satsu et al., 2013). While not much is known so far concerning the biological effects of CYM-5520, the related compound CYM-5478 (Satsu et al., 2013) reduced cisplatin-mediated cell death by reducing reactive oxygen species (ROS) in C6 glioma cells (Herr et al., 2016). Taken together, further development is required in the area of S1P_2_ receptor pharmacology.

## Anti-Migratory Effect of S1P_2_ and Consequences for Angiogenesis and B Cell Homing

One of the main effects of the S1P_1_ receptor is induction of migration and chemotaxis within gradients of S1P (Spiegel et al., 2002). In contrast to S1P_1_, S1P_2_ inhibits migration in many cell types, including vascular endothelial and smooth muscle cells as well as tumor cells, via activation of Rho and inhibition of Rac (Okamoto et al., 2000; Arikawa et al., 2003; Goparaju et al., 2005; Lepley et al., 2005; Tamama et al., 2005; Takashima et al., 2008). The S1P_2_ antagonist JTE-013 augmented S1P-induced angiogenesis *in vivo* in a Matrigel implant assay (Inoki et al., 2006). The S1P_2_ receptor appears to be upregulated in senescent endothelial cells, such as pulmonary microvascular endothelial cells from aged rats, and senescence-associated impairment in chemotaxis and migration was attenuated by down-regulation of S1P_2_ (Lu et al., 2012). In human umbilical vein endothelial cells, the S1P_2_ receptor suppressed angiogenic sprouting via a Gα_12/13_/leukemia-associated RhoGEF (LARG)/RhoC-signaling cascade (Del Galdo et al., 2013). Nevertheless, both pro- and anti-migratory S1P receptors, i.e., S1P_1-3_, are required for proper development of the vasculature during embryonic development (Kono et al., 2004). In the mouse retina, S1P_2_ was not required for normal angiogenesis (Skoura et al., 2007). Interestingly, S1P_2_ was induced by hypoxia, and its knockout reduced the hypoxia-associated pathologic neovascularization in the vitreous chamber, while it augmented the revascularization into the avascular zones of the retina (Skoura et al., 2007). While these observations suggest that antagonism at S1P_2_ might be useful for treatment of pathologic neovascularization, S1P_2_ knockout in fact went along with enhanced tumor angiogenesis (Du et al., 2010), suggesting that this indication should be regarded with caution.

Interestingly, similar to the pro-migratory effect of the S1P_1_ receptor, which is required for lymphocyte egress from secondary lymphatic tissues, the anti-migratory effect of the S1P_2_ receptor plays an eminent role in the regulation of lymphocyte localization. By inhibition of migration and proliferation, S1P_2_ mediates the confinement of B cells and follicular T helper cells to lymph node germinal centers (Green et al., 2011; Moriyama et al., 2014). It is likely a consequence thereof that mice lacking the S1P_2_ receptor develop clonal B cell lymphomas with age (Cattoretti et al., 2009). Furthermore, somatic mutations in the 5′ sequence of the S1PR2 gene were detected in about 25% of human germinal center-derived diffuse large B cell lymphomas (Cattoretti et al., 2009). Importantly, mice which were heterozygous for the S1P_2_ receptor did not develop lymphomas (Cattoretti et al., 2009), suggesting that treatment with a pharmacological inhibitor would probably not cause this devastating condition, although this is clearly one of the critical facets in S1P_2_ antagonist development.

## Contractile Effect of S1P_2_ in Smooth Muscle and Consequences for Vessel Tone, Blood Flow, and Inner Ear Function

The S1P_2_ receptor induces contraction of diverse types of smooth muscle, including vascular, bronchial, intestinal, and bladder smooth muscle, by inducing increases in intracellular Ca^2+^ concentration and activation of Rho/Rho kinase (Ohmori et al., 2003; Hu et al., 2006; Kendig et al., 2013). In the vascular system, endothelial S1P_1_ and S1P_3_ receptors mediate NO-dependent vasodilation, while smooth muscle S1P_2_ and S1P_3_ receptors mediate vasoconstriction, with differential responses in different vascular beds (Igarashi and Michel, 2009; Kerage et al., 2014). S1P_2_ knockout mice had normal mean arterial pressure and left ventricular function, but reduced renal and mesenteric vascular resistance which went along with an enhanced blood flow in the affected areas (Lorenz et al., 2007). Furthermore, the S1P_2_ receptor contributed to blood pressure elevation and vasoconstriction after α-adrenergic stimulation (Lorenz et al., 2007). In a mouse model of myocardial infarction-induced heart failure, S1P_2_ knockout mice did not develop enhanced myogenic responses (Hoefer et al., 2010). While blood flow in cerebral arteries was dependent on S1P_3_ (Salomone et al., 2008), S1P_2_ appears to play a role in perfusion of lung, liver, and kidney. Thus, S1P_2_ activation increases pulmonary vascular resistance in isolated perfused mouse lung (Szczepaniak et al., 2010). Another study suggested that S1P-induced pulmonary vasoconstriction was mediated by S1P_4_ (Ota et al., 2011), but it is a contradiction that JTE-013, as a S1P_2_/S1P_4_ antagonist (Long et al., 2010), had no effect in that study. Interestingly, hypoxia-induced pulmonary hypertension in rodents was reduced by JTE-013 (Chen et al., 2014). In agreement, S1P_2_ antagonists have been suggested for the treatment of pulmonary hypertension (Xing et al., 2015). In isolated perfused rat liver, S1P_2_ activation increased portal pressure (Ikeda et al., 2004), and S1P_2_ antagonism reduced portal vein pressure in cirrhotic rats after bile duct ligation (Kageyama et al., 2012). Finally, S1P_2_-mediated vasoconstriction in the isolated perfused kidney was enhanced in diabetic rats (Bautista-Pérez et al., 2011).

While these results suggest that S1P_2_ antagonism could be helpful to increase organ blood flow, there is also a caveat: S1P_2_ knockout mice are deaf and have a progressive loss of vestibular function (MacLennan et al., 2006). Among the earliest lesions in the cochlea of S1P_2_-deficient mice, there were defects in the stria vascularis (Kono et al., 2007). These data suggest that the absence of S1P_2_ caused a dysregulation of the spiral modiolar artery tone, and subsequently an abnormal perfusion of the inner ear, which is accountable for the phenotype (Kono et al., 2007). In addition, the S1P_2_ receptor also contributes to cochlear hair cell maintenance and survival (Herr et al., 2007, 2016). Thus, it was shown recently that ROS accumulated in the cochlea of S1P_2_ knockout mice (Herr et al., 2016). Furthermore, S1P_2_ inhibited NADPH oxidase-3 activity in a recombinant system, and the S1P_2_ agonist, CYM-5478, reduced cisplatin-induced ROS formation in C6 glioma cells (Herr et al., 2016). Also very recently, the S1P_2_ receptor gene has been allocated to the autosomal-recessive non-syndromic hearing impairment locus DFNB68 on chromosome 19, and two pathogenic S1P_2_ variants have been identified (Santos-Cortez et al., 2016). In conclusion, S1P_2_ receptor agonists might be a treatment option for cisplatin- or gentamicin-induced ototoxicity (Nakayama et al., 2014; Herr et al., 2016), while S1P_2_ antagonists will have to be analyzed for potential ototoxic effects.

## Vascular Permeability and Vascular Inflammation

While early studies reported that functional antagonists at S1P_1_ improved the vascular endothelial barrier, e.g., in lipopolysaccharide (LPS)-induced edema, it soon turned out that this was due to the initial agonistic activity of the compounds, which was however followed by an increase in vascular permeability on the long term (Xiong and Hla, 2014). Here again, the S1P_2_ receptor plays an opposite role, as activation of S1P_2_ causes a disruption of endothelial adherens junctions and increased paracellular permeability. Consequently, JTE-013 improved barrier integrity (Sanchez et al., 2007). An improvement of the vascular barrier by knockout or inhibition of S1P_2_ has been shown in endothelial cell cultures and in diverse *in vivo* models including stroke (Kim et al., 2015), experimental autoimmune encephalitis (EAE; Cruz-Orengo et al., 2014), and anaphylactic shock (see below). Thus, JTE-013 attenuated the H_2_O_2_-evoked vascular leak in the isolated perfused rat lung (Sanchez et al., 2007). LPS and tumor necrosis factor-α (TNF-α) upregulated S1P_2_ in endothelial cells. Importantly, vascular inflammation induced by LPS or TNF-α, with NF-κB activation and upregulation of vascular cell adhesion molecule (VCAM)-1, intercellular adhesion molecule (ICAM)-1, E-selectin, and monocyte chemoattractant protein (MCP)-1, was reduced by inhibition and/or knockout of S1P_2_ (Du et al., 2012; Zhang W. et al., 2013). In models of LPS-induced acute lung injury or endotoxemia, both vascular and alveolar leakage were reduced by these pharmacological or genetic approaches (Sammani et al., 2010; Zhang G. et al., 2013). Taken together, a barrier disruptive role of the S1P_2_ receptor, and thus a therapeutic potential for S1P_2_ antagonists in diverse conditions of vascular barrier breakdown, is supported by a broad range of studies. However, it was also shown that S1P_2_ might be protective against acute vascular barrier disruption in active anaphylaxis and after injection of platelet-activating factor (Cui et al., 2013; see below). These authors observed that the activities of Akt and endothelial NO synthase were enhanced in S1P_2_ knockout aorta, lung, and endothelial cells, and the enhanced NO release in turn compromised vascular barrier function (Cui et al., 2013). Furthermore, S1P_2_ contributed to maintenance of adherens junctions in this study, in contrast to previous reports (Sanchez et al., 2007). The reason for this, however, remains unclear.

## Atherosclerosis

S1P occurs at high concentrations in blood plasma and serum, where it is bound mostly to high-density lipoproteins and albumin, and both pro- and anti-atherosclerotic activities of S1P have been described (Levkau, 2015). The S1P_2_ receptor, in particular, is significantly upregulated by inflammatory mediators in the endothelium, and it mediates vascular inflammation (see above), which might be a key factor for promoting atherosclerosis. In fact, S1P_2_ is upregulated in atherosclerotic endothelium (Estrada et al., 2008). However, after carotid artery ligation, S1P_2_-deficient mice developed large neointimal lesions, which went along with medial and intimal smooth muscle proliferation (Shimizu et al., 2007), suggesting that S1P_2_ on vascular smooth muscle cells was atheroprotective. In agreement, S1P_2_, by inducing serum response-factor enrichment of CArG box promotor regions, induced phenotypic modulation of smooth muscle cells in response to S1P (Wamhoff et al., 2008). On the other hand, atherosclerosis in the mouse aorta was decreased by S1P_2_ knockout in apolipoprotein E-deficient mice (Skoura et al., 2011). This study showed that body weight, plasma cholesterol, plasma triglyceride, and lipoprotein profiles were not altered in atherosclerotic S1P_2_ knockout mice, but that the number of macrophages and foam cells infiltrating the vessel wall was decreased, and that S1P_2_ in the myeloid cells was responsible for the effect. It was concluded that S1P_2_ signaling retained macrophages in the plaques, where they promoted inflammation (Skoura et al., 2011). Recently, it was shown that a knockout of both Gα_12_ and Gα_13_ in myeloid cells protected LDL receptor-deficient mice from atherosclerosis, which is of importance here, since the S1P_2_ receptor was identified as the major G_12/13_ activator in the relevant cells, which were identified as peritoneal macrophages (Grimm et al., 2016). In brief, the authors showed that the alternative, anti-inflammatory polarization of aortic macrophages in those mice was an indirect effect, mediated by activation of atheroprotective B cells through classical, pro-inflammatory peritoneal macrophages. Importantly, in both bone marrow-derived and peritoneal macrophages from myeloid G_12/13_ knockout mice, pro-inflammatory cytokines were strongly upregulated, and this was mimicked by JTE-013 in wild-type macrophages (Grimm et al., 2016). Thus, the study suggested that G_12/13_, and S1P_2_ as major G_12/13_ activator in macrophages, decreased rather than increased pro-inflammatory cytokine production, but nevertheless, atherosclerosis was attenuated by inhibition of this pathway (Grimm et al., 2016). Taken together, inhibition of S1P_2_ might be atheroprotective, but further studies on the underlying mechanisms are clearly required.

## Mast Cells and Anaphylaxis

Early reports have shown that in mast cells, the S1P_2_ receptor is involved in an autocrine loop that augments degranulation induced by Fc𝜀 receptor-I crosslinking (Jolly et al., 2004). However, recent studies on the role of S1P_2_ in anaphylaxis yielded contradictory results (**Table 1**): One group observed that JTE-013 and S1P_2_ knockout attenuated the severity of anaphylaxis and reduced circulating histamine levels as well as pulmonary edema and allergic lung infiltration in a mouse model of immunoglobulin E (IgE)/antigen-triggered anaphylaxis (Oskeritzian et al., 2010, 2015). Interestingly, after histamine injection to shortcut mast cell degranulation, JTE-013 and S1P_2_ knockout did not attenuate the anaphylactic response, suggesting that S1P_2_ acted primarily by augmenting histamine release from mast cells during the onset of anaphylaxis (Oskeritzian et al., 2010). Another group observed that the symptoms of histamine-induced anaphylaxis were more severe in S1P_2_ knockout mice (Olivera et al., 2010). Most importantly, they observed a poor recovery after both histamine and IgE/antigen challenge in these mice, which went along with a severe hypotension and a delay in plasma histamine clearance (Olivera et al., 2010, 2013b). Yet another group even questioned that S1P_2_ knockout improved the vascular barrier breakdown, as deletion of this receptor aggravated the vascular leak and strongly impaired survival in mice challenged with antigen or platelet-activating factor (Cui et al., 2013). As possible explanations for the discrepant findings, differential responses of mast cell populations, the respective activity of the IgE preparations, active versus passive anaphylaxis as well as a role of the mouse strains were considered (Cui et al., 2013; Olivera et al., 2013b; Oskeritzian et al., 2015). Taken together, it remains open whether the potential beneficial effects of S1P_2_ antagonists, i.e., inhibition of mast cell degranulation, improvement of the vascular barrier, and attenuation of airway constriction, would outweigh their potential deleterious effect, i.e., aggravation of hypotension, in human anaphylaxis.

**Table 1:** Overview of controversial publications on the role of the S1P_2_ receptor in anaphylaxis, inflammation and cancer.

Anaphylaxis	Aggravation of anaphylaxis by S1P_2_	Attenuation of anaphylaxis by S1P_2_	Comments
	Jolly et al., 2004		S1P_2_ augments degranulation by Fc𝜀 receptor-I.
	Oskeritzian et al., 2010, 2015		S1P_2_-KO and JTE-013 reduce histamine release and attenuate severity of anaphylaxis.
		Olivera et al., 2010, 2013b	Severe hypotension and delayed plasma histamine clearance in S1P_2_-KO.
		Cui et al., 2013	Impaired survival in S1P_2_-KO mice challenged with antigen; only study to show vascular leak in S1P_2_-KO.

**Inflammation**	**Pro-inflammatory role of S1P_2_**	**Anti-inflammatory role of S1P_2_**	**Comments**

	Siehler et al., 2001; Blom et al., 2010; O’Sullivan et al., 2014		S1P_2_ activates NF-κB.
	Damirin et al., 2005; Li et al., 2009; Völzke et al., 2014		S1P_2_ induces COX-2 expression and PG synthesis.
	Skoura et al., 2011		S1P_2_-KO and JTE-013 reduce cytokine levels in LPS-treated mice.
		Stradner et al., 2013	S1P_2_ counteracts IL-1β in chondrocytes and cartilage.
	Sammani et al., 2010		S1P_2_-KO reduces bronchial leakage in LPS-induced lung injury.
	Roviezzo et al., 2007; Trifilieff et al., 2009; Fuerst et al., 2014		These studies show a principle role for S1P_2_ in allergic airway inflammation and constriction.
	Chiba et al., 2010		S1P_2_ is downregulated during sensitization, resulting in a loss of bronchoconstriction by S1P.
		Michaud et al., 2010	S1P_2_-KO increases number of peritoneal macrophages in thioglycollate-induced peritonitis.
	Roviezzo et al., 2011		JTE-013 reduces cell recruitment after subcutaneous injection of zymosan.

**Cancer**	**Pro-cancerous role of S1P_2_**	**Anti-cancerous role of S1P_2_**	**Comments**

		Cattoretti et al., 2009	B cell lymphoma in homozygous S1P_2_-KO mice.
		Yamaguchi et al., 2003	S1P_2_ overexpression and i.p. application of S1P reduce lung metastasis of implanted B16 melanoma cells.
		Du et al., 2010	Accelerated tumor growth in S1P_2_-KO mice.
	Young and van Brocklyn, 2007; Salas et al., 2011; Ponnusamy et al., 2012; Kawahara et al., 2013; Bi et al., 2014		Role for S1P_2_ in metastasis and/or chemoresistance of diverse tumors and/or tumor cell lines
	Miller et al., 2012; Yu et al., 2012; Adada et al., 2015		Activation of pro-cancerous signaling pathways by S1P_2_
	Li et al., 2015		Anti-tumor effect of an S1P_2_ antagonist

*KO, knockout. This table does not claim to be exhaustive.*

## Inflammation and Fibrosis

As mentioned above, the S1P_2_ receptor plays a role in vascular permeability and inflammation, atherosclerosis, mast cell function, and anaphylaxis. Further studies suggest that the S1P_2_ receptor acts pro-inflammatory also in other inflammatory settings. In LPS-treated mice, elevated serum levels of interleukin (IL)-1β and IL-18 were reduced by S1P_2_ knockout and JTE-013 (Skoura et al., 2011). In diverse cell types, S1P_2_ activated NF-κB (Siehler et al., 2001; Blom et al., 2010; O’Sullivan et al., 2014) or induced COX-2 expression and PG synthesis (Damirin et al., 2005; Li et al., 2009; Völzke et al., 2014). As mentioned above, in a mouse model of LPS-induced lung injury, knockout of S1P_2_ reduced protein leakage into the bronchoalveolar lavage fluid (Sammani et al., 2010). On the other hand, in primary human chondrocytes and osteoarthritis cartilage, S1P_2_ counteracted the pro-inflammatory signaling of IL-1β, including IL-1β-induced upregulation of inducible NO synthase (Stradner et al., 2013).

S1P generally induces airway smooth muscle contraction (Rosenfeldt et al., 2003) and plays a role in airway hyper-responsiveness and asthma (Kume et al., 2007; Roviezzo et al., 2010, 2015). Recently, it was shown that S1P repressed β_2_ adrenergic activity in airway smooth muscle cells by increasing COX-2-mediated PGE_2_ production (Rumzhum et al., 2016). S1P_2_ and S1P_3_ appear to be involved in these effects (for review, see Lai et al., 2011). Thus, JTE-013 inhibited ovalbumin-induced contraction of lung parenchymal strips from ovalbumin-sensitized rats (Trifilieff et al., 2009). However, in isolated bronchial rings, S1P augmented KCl-induced contraction in a JTE-013-sensitive manner only in bronchi from control mice, not from antigen-challenged mice, which was traced back to a down-regulation of S1P_2_ during sensitization (Chiba et al., 2010). In contrast, S1P_2_ and S1P_3_ were upregulated during ovalbumin-sensitization in the study of Roviezzo et al. (2007). Importantly, S1P_2_ and S1P_3_ receptors induced a steroid-resistant pro-remodeling response in airway smooth muscle cells (Fuerst et al., 2014). S1P_2_ furthermore had a pro-inflammatory influence on the bronchial epithelium, as S1P-induced IL-8 release via NF-κB was inhibited by JTE-013 in human bronchial epithelial cells (O’Sullivan et al., 2014). The data suggest that S1P_2_ antagonists might have a beneficial effect on airway inflammation and remodeling in asthma.

Notably, the inhibitory influence of S1P_2_ on migration plays an important role in the recruitment of inflammatory cells to sites of inflammation. Thus, in thioglycollate-induced peritonitis, knockout of the S1P_2_ receptor led to an increase in the number of macrophages in the peritoneal cavity (Michaud et al., 2010). On the other hand, cell recruitment after subcutaneous injection of zymosan was reduced by JTE-013 (Roviezzo et al., 2011). These seemingly contradictory observations suggest that the underlying migratory events of the different cell types are regulated in a complex manner by S1P_2_.

Some additional activities of the S1P_2_ receptor might be important in inflammation. Thus, S1P_2_ reduced antigen capture by murine Langerhans cells, thereby potentially alleviating cutaneous contact hypersensitivity (Japtok et al., 2012). S1P_2_ furthermore suppressed macrophage phagocytosis, thereby impairing host defense in sepsis (Hou et al., 2015). Finally, while knockout and blockade of S1P_2_ improved the tightness of blood–brain barrier and ameliorated EAE, the receptor was found to be more abundant in female EAE mice and female patients with multiple sclerosis compared to their male counterparts, and it was suggested that this underlies the enhanced female CNS autoimmune susceptibility (Cruz-Orengo et al., 2014). Taken together, we are just beginning to understand the complex role of S1P_2_ in infection, inflammation, and autoimmunity.

With regard to fibrosis, it has been shown that intracellular S1P acts anti-fibrotic, while extracellular S1P has a pro-fibrotic activity. However, the respective role of the different S1P receptor subtypes appears to be less clear (Schwalm et al., 2013). In bleomycin-induced lung injury, a prolonged exposure to S1P_1_ functional antagonists including fingolimod not only worsened the vascular barrier function but also increased the fibrotic response (Shea et al., 2010), suggesting that S1P_1_ acts anti-fibrotic in pulmonary fibrosis. On the contrary, pro-fibrotic events such as Smad activation, induction of connective tissue growth factor (CTGF), and enhanced synthesis of extracellular matrix components, were dependent on signaling pathways involving G_12/13_ and/or Rho/Rho kinase, thereby pointing toward S1P_2_ and S1P_3_ as the main pro-fibrotic S1P receptors (Schwalm et al., 2013). Indeed, in carbon tetrachloride-induced liver fibrosis, S1P_2_ knockout mice showed reduced accumulation of hepatic myofibroblasts and decreased induction of fibrotic markers (Serriere-Lanneau et al., 2007; Ikeda et al., 2009). Considering also the role of S1P_2_ in portal hypertension (Kageyama et al., 2012; see above), S1P_2_ antagonists may turn out to be helpful in liver fibrosis. Other reports suggest a role for S1P_2_ and/or S1P_3_ in pulmonary, renal, or cardiac fibrosis (Schwalm et al., 2013). Of note, also the S1P_5_ receptor appears to be involved in fibrosis, as it has recently been shown to induce CTGF expression in renal mesangial cells (Wünsche et al., 2015).

## Diabetes Mellitus and Insulin Resistance

Function and survival of pancreatic β-cells, as well as the responsiveness of insulin-sensitive tissues to insulin, play an important role in diabetes mellitus, and both are regulated by the SphK/S1P axis (Jessup et al., 2011; Fayyaz et al., 2014b). While earlier work showed that S1P itself contributed to glucose-stimulated insulin secretion and generally improved β-cell survival (Fayyaz et al., 2014b), it was shown recently that the S1P_2_ receptor had a rather negative impact on pancreatic β-cells. In streptozotocin-induced diabetes mellitus, S1P_2_ knockout mice had lower blood glucose levels, higher insulin/glucose ratios, less β-cell apoptosis, and a better survival rate (Imasawa et al., 2010). Furthermore, JTE-013 attenuated the development of diabetes in streptozotocin-treated wild type mice (Imasawa et al., 2010). In New Zealand obese mice, plasma concentrations of S1P increased over 28 days of high-fat diet, and treatment with JTE-013 reduced the concomitant β-cell loss (Japtok et al., 2015). Mechanistically, S1P_2_ counteracted the anti-apoptotic and proliferative effects of insulin by inhibiting Akt (Japtok et al., 2015).

Of the insulin-sensitive tissues, both skeletal muscle and hepatocytes were regulated by S1P_2_ (Rapizzi et al., 2009; Fayyaz et al., 2014a). In C2C12 myoblasts, S1P caused a ROS-dependent transphosphorylation of the insulin receptor, thereby increasing glucose uptake into the cells. S1P-induced ROS production was inhibited by JTE-013, suggesting that it was the S1P_2_ receptor which mimicked the activity of insulin in myoblasts (Rapizzi et al., 2009). In hepatocytes, however, S1P induced insulin resistance as it attenuated insulin-stimulated Akt phosphorylation in primary rat and human hepatocytes. This effect was sensitive to JTE-013, indicating an involvement of S1P_2_ which was the prevailing S1P receptor subtype on the mRNA level in hepatocytes. Importantly, in New Zealand obese mice, JTE-013 attenuated the continuous increase in blood glucose levels under high-fat diet, and partially reversed the high-fat diet-mediated loss in phospho-Akt in the liver of these mice (Fayyaz et al., 2014a). Thus, insulin sensitivity was regulated by S1P_2_ in a different manner in skeletal muscle and hepatocytes, respectively. Finally, it has to be mentioned that not much is known about S1P_2_ receptor activity in adipocytes, but a recent study suggests that the receptor had an anti-adipogenic effect in 3T3-L1 preadipocytes, as it decreased the expression of peroxisome proliferator-activated receptor-γ (PPAR-γ), CCAAT/enhancer binding protein-α and adiponectin, and decreased the triglyceride content of the cells (Moon et al., 2015). Consequently, the authors concluded that activation of S1P_2_ might be helpful against obesity (Moon et al., 2015). However, one has to keep in mind, that activation of PPAR-γ is well-known to improve insulin sensitivity and plasma lipid profiles, and to inhibit inflammation (Yki-Jarvinen, 2004; Cariou et al., 2012). Thiazolidinediones, which are PPAR-γ agonists, have been used for years as insulin sensitizers in diabetes mellitus type 2, but they have been gradually removed from the market because of hepatotoxicity, increased cardiovascular risk, or increased occurrence of bladder cancer (Yki-Jarvinen, 2004; Cariou et al., 2012). Presently, pharmacologists aim at next-generation PPAR-γ agonists with improved safety profiles (Sauer, 2015). In this context, it will be interesting to study the potential of S1P_2_ antagonists as inducers of PPAR-γ in relation to the reported negative effects of thiazolidinediones.

## Skeletal Muscle

The SphK/S1P axis plays an important role in skeletal muscle (patho)physiology, and the S1P_2_ receptor, in particular, is involved in myogenic differentiation and muscle regeneration (Donati et al., 2013). Thus, by antisense, silencing or overexpression approaches, it was shown that S1P_2_ induced differentiation of C2C12 myoblasts and contributed to myogenesis by low doses of TNF-α (Donati et al., 2005, 2007). Importantly, S1P_2_ knockout mice had no alteration in the soleus muscle mass and morphology, however, as their body weight increased during aging stronger than that of wild-type mice, their muscle/body weight ratio decreased more strongly (Germinario et al., 2012). In a model of soleus muscle degeneration by the myotoxic compound notexin, knockout of or antagonism at S1P_2_ delayed muscle regeneration and decreased the expression of the promyogenic marker myogenin (Germinario et al., 2012). It was finally shown that S1P_2_ activated quiescent satellite cells and facilitated muscle regeneration via STAT3 (signal transducer and activator of transcription-3; Saba and de la Garza-Rodea, 2013). Taken together, it will be important to analyze the impact of a long-term treatment with an S1P_2_ antagonist on skeletal muscle homeostasis.

## Cancer

It appears obvious that inhibition of a receptor which acts anti-migratory and often anti-proliferative would result in enhanced tumorigenesis and particularly in metastasis. Indeed, lack or mutation of S1P_2_ is linked to B cell lymphoma, as explained above (Cattoretti et al., 2009). In an early study with B16 melanoma cells which endogenously express only S1P_2_, pretreatment of the cells with S1P before implantation as well as daily i.p. application of S1P reduced lung metastasis, and stable overexpression of S1P_2_ enhanced this effect (Yamaguchi et al., 2003). Remarkably, this study not only shows an anti-metastatic role of S1P_2_, but also contradicts the anti-tumor effect of anti-S1P-antibody treatment reported in many studies (e.g., Ader et al., 2015, for review see Sabbadini, 2011). Furthermore, lung carcinoma and melanoma cells implanted into S1P_2_ knockout mice showed accelerated tumor growth, enhanced angiogenesis and more efficient recruitment of CD11b-positive bone marrow-derived cells into the tumors (Du et al., 2010). However, several reports underline a rather pro-cancerous role of S1P_2_ (**Table 1**): although S1P_2_ inhibited migration of glioma cells, it enhanced the expression of the matricellular protein CCN1/Cyr61 and stimulated glioma cell invasiveness and adhesion (Young and van Brocklyn, 2007). In chronic myeloid leukemia, S1P_2_ enhanced Bcr–Abl1 stability via inhibition of protein phosphatase-2a, and inhibition of SphK1/S1P_2_ restored the chemosensitivity of leukemia allografts in mice (Salas et al., 2011). S1P_2_ also increased the chemoresistance of colon carcinoma cells (Kawahara et al., 2013). Inhibition of S1P_2_ upregulated breast carcinoma metastasis suppressor-1, which in turn suppressed lung metastasis of bladder carcinoma cells (Ponnusamy et al., 2012). Furthermore, pancreatic cancer cells implanted together with S1P_2_ receptor-deficient pancreatic stellate cells showed less cancer growth and metastasis *in vivo* (Bi et al., 2014). Moreover, the above-mentioned phosphorylation of ERM proteins mediated by S1P_2_ was suggested to take part in cancer cell invasion (Adada et al., 2015). Finally, S1P_2_-mediated activation of the Hippo pathway might be pro-cancerous (Miller et al., 2012; Yu et al., 2012; see above). As metastasis of epithelium-derived tumors has been linked to basal rather than apical epithelial extrusion, it is interesting to note that the S1P_2_ antagonist, JTE-013, preferentially inhibited apical extrusion (Gu et al., 2011), although the proposed role of reduced S1P production in basal extrusion of oncogenic K-Ras-expressing cells remains unclear as S1P production by these cells has not been quantified (Slattum et al., 2014). Taken together, apart from the specific situation with S1P_2_ in B-cell lymphoma, the presently available studies suggest that the S1P_2_ receptor controls mechanisms which both promote and prevent tumor growth, invasion, and metastasis. Therefore, for development of S1P_2_ antagonists, thorough studies with the development candidates are required to address their potential for promotion of tumor growth and metastasis.

## Conclusion

As described in the present review, the S1P_2_ receptor plays an important role in many tissues and organs, and therefore, a therapeutic application of S1P_2_ antagonists will necessarily cause unintended effects. Here, an attempt is made to suggest potential therapeutic indications for S1P_2_ antagonists, and to point out the potential risks that have to be taken into account during drug development. First of all, the available data suggest that selective S1P_2_ receptor antagonists might be helpful in pulmonary hypertension, chronic obstructive pulmonary disease, and lung fibrosis. Similarly, portal hypertension and liver fibrosis, which often occur in conjunction, might be an indication. Although S1P_2_ antagonists will probably improve the vascular endothelial barrier, they are likely to cause problems in conditions with critical systemic arterial hypotension, such as sepsis or anaphylaxis. Finally, an interesting approach will be to test the therapeutic potential of S1P_2_ antagonists in diabetes mellitus type 2 with insulin resistance and progressive β-cell loss, and to see whether atherosclerosis is also attenuated in these patients. As the proposed indications require a chronic treatment, the potential long-term adverse effects need to be carefully addressed. Potentially devastating effects in the central nervous system, such as neuronal hyperexcitability, seizures, memory deficits, and increased anxiety (MacLennan et al., 2001; Akahoshi et al., 2011), suggested by observations with S1P_2_ knockout mice or JTE-013 treatment, must be avoided by appropriate chemical–pharmaceutical engineering, i.e., by designing compounds that do not cross the blood brain barrier. Other critical points are inner ear functionality and B cell lymphoma. However, a full receptor knockout cannot be compared to the activity that can be reached with a (competitive) receptor antagonist. In this regard, it is important that mice that were heterozygous for the S1P_2_ receptor did not develop B cell lymphoma, and that hearing impairment due to S1P_2_ missense mutations in humans was autosomal-recessive. While the potential of S1P_2_ antagonists to induce or exacerbate tumor growth and metastasis has to be addressed carefully, these agents might also act protective in cancer. For instance, in a recent study with a novel S1P_2_ antagonist, which is a derivative of JTE-013 with improved properties, the compound inhibited the growth of neuroblastoma xenografts (Li et al., 2015).

Indeed, a major problem in this research area is the lack of appropriate tools. As discussed above, the widely used S1P_2_ antagonist, JTE-013 (Osada et al., 2002), has a low potency and lacks selectivity and stability *in vivo* (Long et al., 2010; Li et al., 2015). Other more recently synthesized S1P_2_ agonists and antagonists still await further characterization (Satsu et al., 2013; Kusumi et al., 2015; Li et al., 2015). In conclusion, the discovery of novel highly selective antagonists with high affinity at S1P_2_ is urgently required to address pending questions regarding the roles and effects of S1P_2_, and to enable definite conclusions with regard to the therapeutic suitability of these drugs.

## Author Contributions

All authors listed, have made substantial, direct and intellectual contribution to the work, and approved it for publication.

## Conflict of Interest Statement

The authors declare that the research was conducted in the absence of any commercial or financial relationships that could be construed as a potential conflict of interest.
